# Overexpression of the *HcPT1.1* transporter in *Hebeloma cylindrosporum* alters the phosphorus accumulation of *Pinus pinaster* and the distribution of HcPT2 in ectomycorrhizae

**DOI:** 10.3389/fpls.2023.1135483

**Published:** 2023-06-15

**Authors:** Laurie Amenc, Adeline Becquer, Carlos Trives-Segura, Sabine D. Zimmermann, Kevin Garcia, Claude Plassard

**Affiliations:** ^1^ Eco&Sols, Univ Montpellier, CIRAD, INRAE, Institut Agro, IRD, Montpellier, France; ^2^ IPSiM, Univ Montpellier, CNRS, INRAE, Institut Agro, Montpellier, France; ^3^ Department of Crop and Soil Sciences, North Carolina State University, Raleigh, NC, United States

**Keywords:** agrotransformation, ectomycorrhizal symbiosis, *Hebeloma cylindrosporum*, immunolocalization, overexpression, phosphate transporter, phosphorus, *Pinus pinaster*

## Abstract

Ectomycorrhizal (ECM) fungi are associated with the roots of woody plants in temperate and boreal forests and help them to acquire water and nutrients, particularly phosphorus (P). However, the molecular mechanisms responsible for the transfer of P from the fungus to the plant in ectomycorrhizae are still poorly understood. In the model association between the ECM fungus *Hebeloma cylindrosporum* and its host plant *Pinus pinaster*, we have shown that the fungus, which possesses three H+:Pi symporters (HcPT1.1, HcPT1.2 and HcPT2), expresses mainly *HcPT1.1* and *HcPT2* in the extraradical and intraradical hyphae of ectomycorrhizae to transport P from the soil to colonized roots. The present study focuses on the role of the HcPT1.1 protein in plant P nutrition, in function of P availability. We artificially overexpressed this P transporter by fungal Agrotransformation and investigated the effect of the different lines, wild-type and transformed ones, on plant P accumulation, the distribution of HcPT1.1 and HcPT2 proteins in ectomycorrhizae by immunolocalization, and 32P efflux in an experimental system mimicking intraradical hyphae. Surprisingly, we showed that plants interacting with transgenic fungal lines overexpressing *HcPT1.1* did not accumulate more P in their shoots than plants colonized with the control ones. Although the overexpression of *HcPT1.1* did not affect the expression levels of the other two P transporters in pure cultures, it induced a strong reduction in HcPT2 proteins in ectomycorrhizae, particularly in intraradical hyphae, but still improved the P status of host plant shoots compared with non-mycorrhizal plants. Finally, 32P efflux from hyphae was higher in lines overexpressing *HcPT1.1* than in the control ones. These results suggest that a tight regulation and/or a functional redundancy between the H+:Pi symporters of *H. cylindrosporum* might exist to ensure a sustainable P delivery to *P. pinaster roots*.

## 1 Introduction

Phosphorus (P) is essential for many functions in plants, such as photosynthesis, energy metabolism or signaling (Raghothama, 2005). Although P is present in many forms in soil, only the free orthophosphate ions H_2_ ${\text{PO}}_{4}^{-}$ and H ${\text{PO}}_{4}^{2-}$ (Pi) can be directly taken up by plants. However, free Pi is often present at very low concentrations (1 to 10 µM) in the soil solution (Hinsinger, 2001). This is due to the low diffusion rate and the high capacity of P anion to complex with cations, making Pi bioavailability a major limiting factor for plant growth in many ecosystems, especially in forests (Plassard and Dell, 2010). To cope with this limited Pi availability, most plant species are able to establish a symbiotic relationship with soil fungi, called mycorrhizal symbiosis. In northern hemisphere forests, although very diverse types of mycorrhizal symbiosis can be found, it is known that trees are predominantly associated with ectomycorrhizal (ECM) fungi (Averill et al., 2019; Becquer et al., 2019; Hackman et al., 2022). These symbiotic fungi form three pseudo-tissues outside and within the host plant roots: the extraradical mycelium that greatly increases the soil volume explored by colonized roots, the fungal mantle that isolates the root from the soil, and the Hartig net formed by intraradical hyphae between plant cortical cells that allows mutualistic exchanges of water and nutrients (Cairney, 2011; Smith et al., 2015; Garcia et al., 2016).

As shown by pioneer experimental studies using ^32^Pi as a tracer, one of the most important benefits of ECM fungi is the enhancement of the host plant P nutrition. Indeed, Melin and Nilson (1950) showed for the first time that ECM hyphae were able to take up ^32^Pi, transfer it to colonized roots, and then radioactive Pi was translocated to the shoot. While Pi uptake from the soil solution relies on the activity of fungal Pi transporters to deliver Pi to the cytoplasm of fungal cells, the molecular mechanisms for Pi efflux from fungal cells into the apoplastic space of mycorrhizae are not yet clearly identified. So far, two main types of high-affinity P transporters have been identified in the yeast *Saccharomyces cerevisiae*: Pi : Na^+^ (ScPho89) and H^+^:Pi (ScPho84) symporters (Bun-Ya et al., 1991; Martinez et al., 1998). Searches for homologous genes in the publicly available genomes of mycorrhizal fungi (Mycocosm, https://mycocosm.jgi.doe.gov/mycocosm/home) revealed that most of ECM basidiomycetes have only genes encoding high-affinity H^+^:Pi symporters, whereas ascomycetes have additional genes encoding for high-affinity Pi : Na^+^ symporters (Casieri et al., 2013; Nehls and Plassard, 2018). In ECM species, only a few proteins have been functionally characterized so far: three P transporters from *Hebeloma cylindrosporum* (HcPT1.1, HcPT1.2 and HcPT2) (Tatry et al., 2009; Becquer et al., 2018a; Becquer et al., 2018b), one from *Boletus edulis* (BePT) (Wang et al., 2014), one from *Rhizopogon luteolus* (RlPT), and one from *Leucocortinarius bulbiger* (LbPT) (Zheng et al., 2016). All these transporters have been characterized as high affinity H^+^:Pi transporters.

In the model fungus *H. cylindrosporum*, the regulation and localization of the three H^+^:Pi transporters have been studied extensively both at the transcript and the protein levels. In pure culture conditions, the gene *HcPT1.1* is upregulated at low Pi (Tatry et al., 2009; Garcia et al., 2013), whereas the expression of the two genes *HcPT1.2* and *HcPT2* seems to be less sensitive to changing P concentrations (Tatry et al., 2009; Becquer et al., 2018b). Altogether, these data suggest that HcPT1.1 is the main H^+^:Pi transporter for P acquisition under P-limiting conditions. In P- sufficient conditions, HcPT1.2 and HcPT2 could ensure most of the P uptake into the fungus. However, the expression level of *HcPT1.2* is very low compared to that of *HcPT2* (Becquer et al., 2018a), suggesting that HcPT2 is the main H^+^:Pi transporter for P uptake when *H. cylindrosporum* is grown in P- sufficient conditions. In symbiotic conditions, HcPT1.1 is specifically localized in the fungal mantle under limited P (Garcia et al., 2013), and HcPT1.2 is only found in the fungal mantle irrespective of the P availability (Becquer et al., 2018b). Recently, we also showed that, although HcPT2 proteins are detected in all parts of the ECM tips (external mycelium, mantle, and Hartig net) irrespective of P availability, the strongest immunolocalization signal of the protein was recorded in the Hartig net of ECM roots grown in P-sufficient soil (Becquer et al., 2018a). Altogether, these results indicate that HcPT1.1 and HcPT1.2 may be preferentially involved in Pi uptake from the soil solution, particularly under P deficiency for HcPT1.1.

Several hypotheses have been proposed so far to explain P efflux from the fungal cells towards the apoplastic space common to the fungal and plant cells in mycorrhizal roots (Ezawa and Saito, 2018; Nehls and Plassard, 2018). Interestingly, Pi and sugar exchanges between fungal and plant cells were simulated *in silico* by Schott et al. (2016) in the arbuscular mycorrhizal symbiosis. The authors proposed that the H^+^:Pi and H^+^:sugar transport systems, as well as the ATPase-proton pumps of both partners function as a network with the following characteristics: 1) the apoplastic concentrations of Pi and sugars is very low (in the micromolar or submicromolar range) and 2) there is always an efflux of Pi from the fungal cell through H^+^:Pi fungal transport systems, and sugar from the root cell to the fungus through the H^+^:sugar transport systems. This modelling of fungus/plant P/C exchange therefore suggests that fungal H^+^:Pi transporters should be able to mediate Pi efflux. This hypothesis supports the results obtained previously with the Pho84 transporter from *S. cerevisiae* by Fristedt et al. (1996). These authors isolated plasma membrane vesicles containing pho84, the protein having an inverted orientation. Despite this inversion, Pho84 mediated the import of Pi into these vesicles, which were hermetically sealed, solely under the dependence of the Pi and H^+^ gradients. These results reinforce the idea that H^+^:Pi symporters could mediate Pi influx and efflux depending on the conditions on either side of the plasma membrane. Additionally, our analysis of transgenic fungal lines either under- or over-expressing *HcPT2*, coupled with ^32^Pi labelling approach, supported a possible efflux function for this transporter in symbiotic conditions (Becquer et al., 2018a).

However, the mechanisms behind P efflux in ECM roots are still debated, as some mycorrhizal fungal species do not possess any HcPT2 orthologs, while HcPT1.1 was consistently found in all the ECM fungal genomes explored so far (Plassard et al., 2019). In the present manuscript, we generated *H. cylindrosporum* lines overexpressing *HcPT1.1* and we quantified their effects on plant P accumulation and the distribution of HcPT1.1 and HcPT2 proteins in ECM cross-sections using specific antibodies. Additionally, we tested the ability of *HcPT1.1*-overexpressing lines to export Pi in a symbiotic interface-mimicking experiment using ^32^Pi as a tracer.

## 2 Materials and methods

### 2.1 Fungal material and production of transgenic lines

We used the wild-type homokaryotic strain h7 of the ECM basidiomycete *H. cylindrosporum* Romagnesi (Debaud and Gay, 1987) to generate transgenic lines *via* agrotransformation, as described previously (Combier et al., 2003; Garcia et al., 2013). To obtain fungal lines overexpressing *HcPT1.1*, we used the vector pPZP-P-T derived from vector pPZP133 harboring the constitutive promoter *gpd* (glyceraldehyde-3-phospho-dehydrogenase) and the cassette *Hc.Sdh* that conferred resistance to carboxin (Ngari et al., 2009). The coding sequence of *HcPT1.1* (accession AJ970313) was amplified from cDNA (Tatry et al., 2009) using specific primers containing *Spe*I restriction sites to facilitate cloning, as described by Becquer et al. (2018a) (**Supplementary Table 1**). After agrotransformation of h7 with the vector overexpressing *HcPT1.1*, we screened the transgenic lines on solid YMG medium (yeast extract 4 g l^-1^, malt extract 10 g l^-1^, glucose 4 g l^-1^) (Rao and Niederpruem, 1969) supplemented with carboxin (0.2 mg l^-1^). Among all lines able to grow on this medium, we selected two of them that we named OE PT1-9 and OE PT1-10. The control line expressing an empty vector was obtained previously (Garcia et al., 2013).

Fungal cultures were maintained in the dark at 25°C on solid YMG medium, supplemented or not with carboxin for the wild-type strain h7 or the three transgenic lines, respectively. Each fungus was also grown in liquid YMG medium for two weeks without shaking prior to RNA isolation.

### 2.2 Yeast transformation and culture

*Saccharomyces cerevisiae* EY917 (MATα *pho84*Δ::*HIS3 pho87*Δ::*CgHIS3 pho89*Δ::*CgHIS3 pho90*Δ::*CgHIS3 pho91*Δ *ADE2*; from E. O’Shea; Wykoff and O’Shea, 2001) was deleted for all the five P transporters but its growth on medium containing galactose was supported by the construction *pGAL-PHO84* inserted in the plasmid pRS314, enabling the cells to express the high affinity H^+^:Pi transporter pho84 (Wykoff and O’Shea, 2001). We transformed EY917 cells, following Dohmen et al. (1991), with the plasmid pFL61 (URA3 marker) containing either the *HcPT1.1* or *HcPT2* coding sequence, as described in Tatry et al. (2009). The cells were plated onto selective solid medium (agarose, 15 g l^-1^) derived from Giots et al. (2003) containing Yeast Nitrogen Base free from NH_4_ and amino acids (5.7 g l^-1^), (NH_4_)_2_SO_4_ (5 g l^-1^), KH_2_PO_4_ (1.36 g l^-1^), L-Leucine (0.38 g l^-1^) and galactose (20 g l^-1^). The medium was supplemented with a mixture of amino acids (1.386 g l^-1^), without L-leucine, L-histidine, L-tryptophan and uracil (**Supplementary Table 2**). Plates were incubated at 30°C and transformed cells appeared 4-5 days later. PCR amplification with primers annealing the plasmid and the gene (**Supplementary Table 1**) was used to check the presence of cDNA in yeast transformants.

To determine yeast growth and P uptake, the cells were cultured in a synthetic liquid medium described in **Supplementary Tables 3** and **4**. To grow *HcPT1.1-* or *HcPT2*-expressing yeasts, the medium contained glucose (20 g l^-1^) and no uracil, whereas untransformed EY917 expressing only *ScPho84* were grown in medium containing galactose (20 g l^-1^) and uracil (50 mg l^-1^).

### 2.3 Ectomycorrhizal plant production

The fungal lines were associated in sterile conditions with maritime pine seedlings (*P. pinaster* Soland in Ait. from the Médoc, Landes-Sore-Vergé source). Seeds were surface-sterilized for 40 min in 37% H_2_O_2_, abundantly rinsed with sterile milli-Q water, and incubated in sterile milli-Q water at 4°C for 48 h. Surface-sterilized seeds were then sown on solid medium (1.5% agar-agar, 0.2% glucose) until germination. Germinated seeds were grown in sterile test tubes and inoculated or not with 3-week-old wild-type or transgenic fungi, as described previously (Plassard et al., 1994; Garcia et al., 2014). 15 ml of sterile mineral nutrient solution (containing in mM: KNO_3_ 0.6, Ca(NO_3_)_2_ 0.2, KH_2_PO_4_ 0.2, KCl 0.2, MgSO_4_, 7H_2_O 1, and 0.5 ml l^-1^ of ferric citrate 1%, 0.2 ml l^-1^ of Morizet and Mingeau (1976) micronutrient solution, 1 ml l^-1^, thiamine 0.1 mg l^-1^) were supplied every week to each plant. All test tubes were placed in a growth chamber under a 16 h/8 h light/dark cycle at 25°C/20°C, 60%/80% relative humidity (RH), CO_2_ concentration of about 350 10^−6^ dm^3^ dm^−3^ and a PAR (Photosynthetically Active Radiation) of approximately 400 μmol m^−2^ s^−1^ (400–700 nm).

After two months in test tubes, plants with visible ectomycorrhizae were used to set up the mycorrhizal experiment, which was carried out in sterile square Petri dishes (12 x 12 cm) with a hole on top to allow the plant shoots to develop outside the dish (Ranoarisoa et al., 2020). For each Petri dish, a soil mixture was prepared in a 50 ml Falcon tube with 10 g of sand (Sigma-Aldrich), 10 g of soil, and 10 ml of P-free mineral nutrient solution (as described above). The soil mixture was sterilized by autoclaving the tubes (115°C, 40 min) twice a week. This temperature was chosen because it kept the Falcon tubes in better condition than 121°C. Also, to control the efficiency of the sterilization under these temperature conditions, we measured soil respiration, which became undetectable only after a second autoclaving, a week after the first one. The soil we used was a chromic cambisol (Cazevielle, Herault, France) with a very low level of bicarbonate-extractable P content (3 mg kg^-1^) and was used either without any treatment (limiting P soil, LP) or after the addition of soluble P (500 mg P kg^-1^ dry soil supplied as KH2PO4; sufficient P soil, SP), as described by Casarin et al. (2004). The soil mixture was poured into the Petri dish together with the sterile water used to rinse the Falcon tube. The dishes were allowed to dry in sterile conditions in a laminar flow cabinet. Then, 10 ml of sterile water were added to rehydrate the soil, and the plant root system was placed on top. The plates were sealed with adhesive tape, weighed, covered with aluminum foil, and placed in the growth chamber with the same conditions as described above. During the growth period (70 days), the moisture level was checked once a week by weighing the dishes and was maintained at its initial level by adding P-free nutrient solution under sterile conditions. At the end of the experiment, six plants were harvested for total P quantification in roots and shoots. The same experiment was repeated twice independently.

### 2.4 Symbiotic interface mimicking experiment

Fungal P efflux was measured using the method we described previously (Becquer et al., 2017; Torres-Aquino et al., 2017; Becquer et al., 2018a). In this experiment, the fungus was previously incubated with KH_2_^32^PO_4_ in liquid medium to follow fungal P movements. The mycelia were then incubated in an interaction medium, free of simple C sources and P (MgSO4 7H_2_O 0.2 mM, CaCl2 0.5 mM, Trizma^®^ base 5 mM, MES hydrate 5 mM, buffered at pH 5.9), mimicking the symbiotic interface of the Hartig net. The experimental setup was a plastic syringe filled with 60 ml of the interaction medium. Each syringe received either only one mycelium or one mycelium and three 2-month-old maritime pine seedlings. The mycelia of wild-type strain h7 and three transgenic lines (Ctrl, OE-PT1-9 and OE PT1-10) were used. Mycelia and plants were incubated for 48 h, with constant aeration. At the end of the incubation, the volume of the syringes and the fresh matter of the mycelia, roots, and shoots of the plants were recorded. The radioactivity was measured in the medium, the plant, and the fungus. All procedures are detailed further in **Supplementary Material 1**.

### 2.5 Quantitative PCR analyses of fungal transformants

After 2 weeks of culture in liquid YMG medium, each mycelium was quickly rinsed in sterile milli-Q water, blotted between sterile filter paper sheets, and placed in liquid nitrogen. Frozen fungal tissues were ground in a mortar with liquid nitrogen, and total RNAs were isolated using the RNeasy Plus Mini kit (Qiagen, Courtaboeuf, France) following the manufacturer’s instructions. Total RNA was quantified using Quant-iT Ribogreen RNA assay kit (Molecular Probes, Invitrogen) and then reverse-transcribed (100 ng per reaction) using the RevertAid H Minus First Strand cDNA synthesis kit (Thermo Fisher Scientific). Two microliters of cDNAs (dilution 1:2) were used for qRT-PCR reactions. Following manufacturer’s recommendations for the LightCycler C1000, CFX96 Real-Time System (Bio-Rad), qRT-PCR mixtures contained SYBR Green Mix (Thermo Scientific) and both forward and reverse primers for amplification of *HcPT1.1, HcPT1.2*, and *HcPT2* (**Supplementary Table 1**) (Protein ID 446637, 448051, 449267, respectively, based on *H. cylindrosporum* h7 genome database (Kohler et al., 2015; Nehls and Plassard, 2018)). Amplifications were performed as follows: 95°C for 15 min; 40 cycles of 95°C for 30 s, 60°C for 1 min; 59°C for 5 s; and finally at 95°C for 5 s. Data were extracted using LightCycler C1000 software. The amount of fungal RNA in each sample was normalized using tubulin (Protein ID 24108) as an internal control (**Supplementary Table 1**). The calculation of gene expression was performed using the measured efficiency for each gene and primer pair as described by Vergne et al. (2007) and expressed as relative expression of four biological replicates and three technical replicates.

### 2.6 Immunolocalization of HcPT1.1 and HcPT2 proteins in yeasts and ectomycorrhizal roots

Antibodies against HcPT1.1 and HcPT2 were produced in rabbits by *Genosphere Biotechnologie France* (www.genosphere-biotech.com). They were raised against peptides corresponding to the N-terminus of each protein, predicted to be outside of the plasma membrane. Those peptides were 5’-MASYQEKGSTEGSQE-3’ for HcPT1.1 (Garcia et al., 2013) and 5’-GDDIEELKKAQKAEEC-3’ for HcPT2 (Becquer et al., 2018a). The original antibodies were used at a dilution of 1:500 (corresponding to 20 μg ml^-1^) for HcPT1.1 and 1:1000 (corresponding to 1.6 µg ml^-1^) for HcPT2, as described previously.

When plants were harvested from the Petri dishes, around 20 ECM root tips per treatment were removed, placed in glass pillboxes, and fixed with paraformaldehyde under a vacuum as described in detail by Cerecetto et al. (2021), except that the vacuum was interrupted every 20 min during the first 2 h incubation at room temperature to improve the penetration of the fixative into the ECM tips. After fixation, ECM root tips were rinsed, dehydrated and embedded in paraffin blocks with around 10 ECM tips per block arranged in several directions to get transversal and longitudinal sections. Each paraffin block was used for one immunolabelling session and two immunolabelling sessions were performed independently. For a given treatment, the procedure of slide preparation is given in **Supplementary Figure 1**. Briefly, each block was cut into 7 μm slices with a microtome (RM2255, Leica Biosystem ). The resulting paraffin slices were placed on 6 silane-treated slides (3 slices/slide) and deparaffinated as described previously (Cerecetto et al., 2021). Thereafter, mycorrhizal root sections were washed three times for 5 min each with PBS buffer and then incubated in 4% bovine serum albumin (BSA) (IgG free) in PBS for 2 h. The BSA was removed and the sections were incubated overnight at 4°C with anti-HcPT1.1 or anti-HcPT2 antibodies diluted in PBS with 4% BSA. The sections were washed five times in PBS and incubated in 4% BSA in PBS containing the goat anti-rabbit IgG Alexa Fluor 488 conjugate (Molecular Probes, Invitrogen™, Carlsbad, CA) (20 μg ml^-1^), used as secondary antibody, for 1 h in the dark. After three washes in PBS, the sections were mounted in Mowiol 4.88 and observed using an epifluorescence microscope (Zeiss AxioImager M2) equipped with a camera AxioCam HRc Rev2. Three images were taken successively for each section: first, the DAPI filter (excitation 359-371 nm, emission ≥ 397 nm) was used to capture the autofluorescence of the fungal tissues and the root endodermis; second, the GFP filter (excitation 450-490 nm, emission 500-550 nm) was used to record the signal from the secondary antibody; and third, the rhodamine filter (excitation 540-552 nm, emission 575-640 nm) captured the autofluorescence of the root tissues (**Supplementary Figure 2**).

Yeast cells grown in synthetic medium were collected after the Pi concentration reached zero for at least 4 hours. A volume of culture medium sufficient to get 12 units of optical density (OD_600nm_) was centrifuged (3000 g, 5 min). The yeast pellet was also fixed with paraformaldehyde using a vacuum, as described by Cerecetto et al. (2021). After rinsing, the cells were incubated directly in BSA 4% for 2 hours. Each yeast solution was then subdivided in four tubes. One tube was used for autofluorescence measurement, two others for immunolabelling with HcPT1 or HcPT2 primary antibodies revealed by the secondary antibodies, and the last one for reaction with secondary antibodies only, following the procedures described for ECM sections. At the last step, yeast cells were suspended in 0.2 ml of phosphate buffered solution (PBS: 5 mM Na_2_HPO_4_ and 130 mM NaCl) and 5 µl were placed on a slide and observed under the epifluorescence microscope. Two images were taken successively, one with the DAPI filter to capture the autofluorescence of the cells and the other one with the GFP filter to capture the immunolabelling of HcPT1.1 and HcPT2 proteins.

### 2.7 Recording of fluorescence levels from immunolabelled ectomycorrhizal sections

Image analysis and merging of images were carried out with Image J software (Abràmoff et al., 2004). After retrieving each image using Bio-format import tool (Linkert et al., 2010; Besson et al., 2019), individual images registered with each filter (**Supplementary Figure 2**) were examined visually to define regions of interest (ROI) first on the DAPI image (**Supplementary Figure 3**) using the following criteria: 1) the region of the central cylinder was clearly defined due to the autofluorescence of the endodermis, 2) the fungal mantle and the mycelium were easily defined from the blue autofluorescence of the fungal cell walls, 3) the region between the central cylinder and the mantle was defined as the Hartig net, and 4) any case of obviously abnormal high fluorescence appearing in the image of the GFP filter was defined as “stain”. The “stain” zone was then cleared in the DAPI image and the pixel intensities of all the zones were recorded using the “measure” function on ImageJ. Defined regions of interest in the ROI manager of ImageJ were then applied to the two other images (GFP and Rhodamine filters in **Supplementary Figure 2**) to extract the corresponding pixel intensities from each zone. All the data were then exported in an excel file to calculate the ratio of intensities between pixels from the GFP filter to those from the DAPI filter, as they represent the intensity of immunolabelling due to the presence of the targeted P transporter in fungal tissues. The value given for the central cylinder was considered as the threshold value for the presence of the transporter. It also serves to compare the two sessions of immunolabelling done on the sections by calculating a correction factor applied to the data if necessary.

### 2.8 Total phosphorus determination in plant tissues and in culture media

Root and shoot tissues were dried at 60°C for 2 days, weighed, and ground. Ten milligrams were mineralized in H_2_SO_4_ (36N) at 330°C as described by Ali et al. (2009). After dilution of the concentrated acid solutions with ultrapure water (1/35, v/v), total P concentrations were determined using the Malachite Green method (Ohno and Zibilske, 1991) twice independently. Concentrations of free Pi in yeast culture medium were also determined using the Malachite Green method.

### 2.9 Statistical analysis

Trancriptomic analyses and plant P accumulation measurements were carried out from 4 and 6 replicates, respectively. Measurements of ^32^P were performed on 6 replicates per condition and per transgenic line. Quantification of HcPT1.1 and HcPT2 signals by image analysis were carried out on 50 to 80 images taken from ectomycorrhizae sampled from plants grown in LP or SP soil. When indicated, differences between means were analyzed by one-way ANOVA followed by Tukey’s HSD *post-hoc* tests. Effect of soil P on HcPT1.1 and HcPT2 levels in ECM sections were analyzed by Student-t-test. Data normality was checked using the Wilk-Shapiro test and, if necessary, the data were either square root or log_10_ transformed prior to the analysis. All statistical analyses were performed with Statistica software (Version 7.1) at the 5% level of statistical significance.

## 3 Results

### 3.1 Expression levels of P transporters in transgenic lines

**Figure 1:**
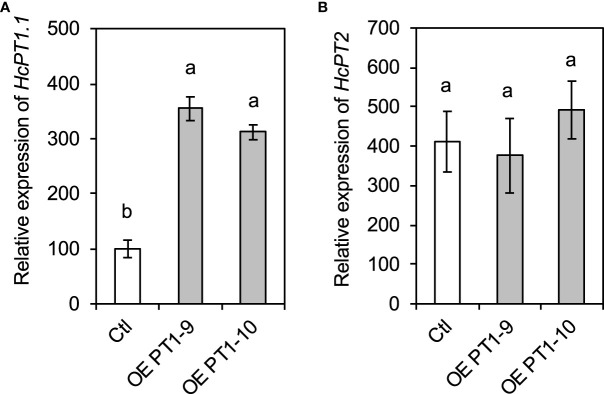
Expression levels of *HcPT1.1* **(A)** and *HcPT2* **(B)** in *HcPT1.1* -overexpressing lines of *H*. *cylindrosporum* grown in pure culture. Gene expression was quantified using RT-qPCR in fungal strains transformed with the empty vector (Ctl) and *HcPT1.1* (OE -PT1-9 and OE -PT1-10). Relative expression was normalized using the a-tubulin housekeeping gene from *H*. *cylindrosporum*. Bars correspond to mean values ± SD (n = 4). Different letters indicate significant differences between means according to one-way ANOVA followed by Tukey’s HSD test (P < 0.05).

The expression levels of *HcPT1.1* and *HcPT2*, the two main H^+^:Pi symporters in *H. cylindrosporum*, were determined by qRT-PCR in the transgenic fungal lines overexpressing *HcPT1.1*. The expression of *HcPT1.1* was significantly enhanced in both overexpressing lines (OE -PT1-9 and OE -PT1-10) by ≥ 250% compared to the control line (**Figure 1A**). In contrast, the expression of *HcPT2* remained similar between the three transgenic lines (**Figure 1B**). Additionally, the overexpression of *HcPT1.1* had no effect on the transcriptional level of the very low expressed homologous gene of *HcPT1.1*, *HcPT1.2* (**Supplementary Figure 4**). These observations indicate that the overexpression of *HcPT1.1* was specific to this particular transporter.

### 3.2 Effect of *HcPT1.1* overexpressing isolates on the P status of *P. pinaster* seedlings

**Figure 2:**
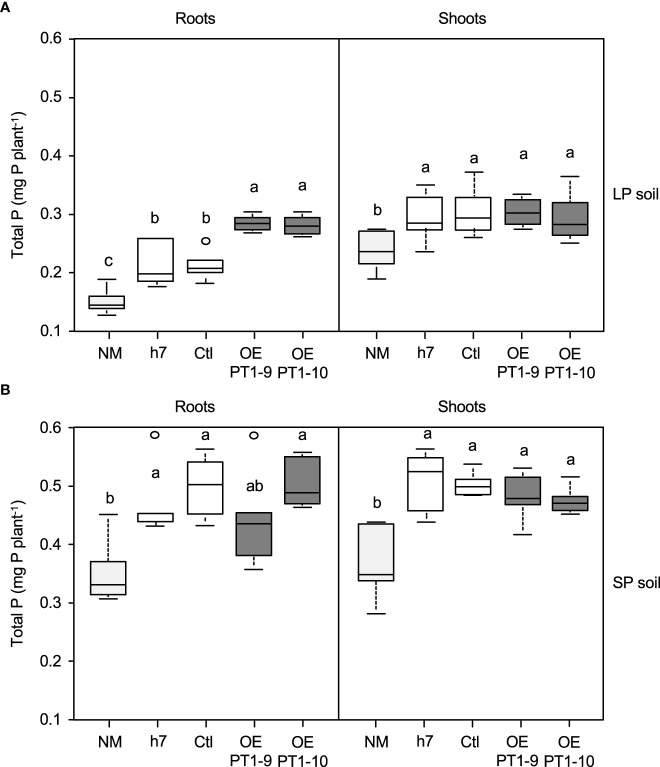
Total phosphorus (P) accumulation in roots and shoots of *P. pinaster* plants grown for 70 d either in poor- (LP soil) **(A)** or enriched- (SP soil) **(B)** P soil. Plants were non-mycorrhizal (NM) or associated with a wild-type *H*. *cylindrosporum* isolate (h7) or transgenic lines transformed with the empty vector (Ctl) or overexpressing *HcPT1.1* (isolates OE PT1-9, OE PT1-10). Box-plots indicate the median, the 1st and 3rd quartiles, and bars give the smallest and highest values of observations (n = 6); empty circles indicate outliers. Different letters indicate significant differences between the fungal lines according to one-way ANOVA followed by Tukey’s HSD test at P < 0.05.

The impact of the *HcPT1.1* overexpressing fungal lines on the P status of host plant seedlings growing in LP and SP soils was quantified by measuring the total P accumulation in roots and shoots (**Figure 2**). In LP soil, the lowest root and shoot P contents were measured in non-mycorrhizal (NM) plants, as expected (**Figure 2A**). Inoculating the seedlings with the wild-type strain h7 or the transgenic control line (Ctl) resulted in similar root and shoot P contents, 33% greater than those of NM plants (**Figure 2A**). This indicates that the transformation of the fungus with the control vector did not affect its ability to allocate P to the host plant, as already observed by Becquer et al. (2018a). However, plants colonized by both *HcPT1.1* overexpressing lines increased the total P in their roots compared to NM plants (+ 100%) and to plants associated with the controls (h7 and Ctl) (+ 50%). In the shoots of these plants, P contents were higher than in non-colonized seedlings (+ 28% for OE PT1-10 and + 12% for OE PT1-9), but similar to plants associated with the control lines, suggesting P retention within the root or even the fungus.

Amending the soil with soluble P (SP soil) increased the amount of P measured in roots (+ 130%) and shoots (+ 40%) of NM plants compared to the LP conditions (**Figure 2B**). Inoculating the seedlings with the control lines significantly increased the P contents in roots (+ 30% on average) and shoots (+ 50% on average) compared to NM plants under the same conditions. Contrary to what was observed in LP soil, the inoculation of both *HcPT1.1* overexpressing lines in SP conditions resulted in similar root and shoot P contents compared to plants colonized by the control fungi.

### 3.3 Effect of *HcPT1.1* overexpressing lines on the distribution of HcPT1.1 and HcPT2 transporters in ectomycorrhizae

**Figure 3:**
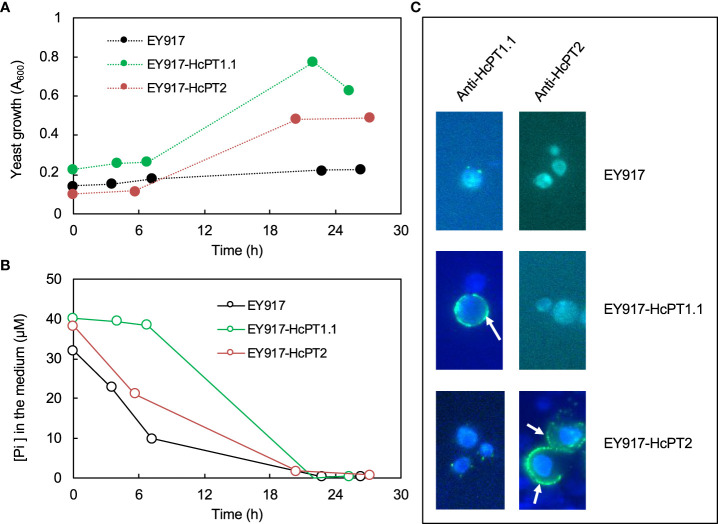
Time-dependent growth **(A)** and Pi decrease in the medium **(B)** when the strain EY917 is expressing pho84 (EY917), HcPT1.1 (EY917-HcPT1.1) or HcPT2 (EY917-HcPT2). Merged images from DAPI and GFP filters **(C)** of yeasts probed with primary antibodies, either anti-HcPT1.1 or anti-HcPT2, and Alexa Fluor 488, showing that HcPT1.1 or HcPT2 were localised at the plasma membrane (white arrows) only in the yeast transformed with the plasmid harbouring *HcPT1.1* or *HcPT2* cDNA. Scale bars correspond to 5 µm.

**Figure 4:**
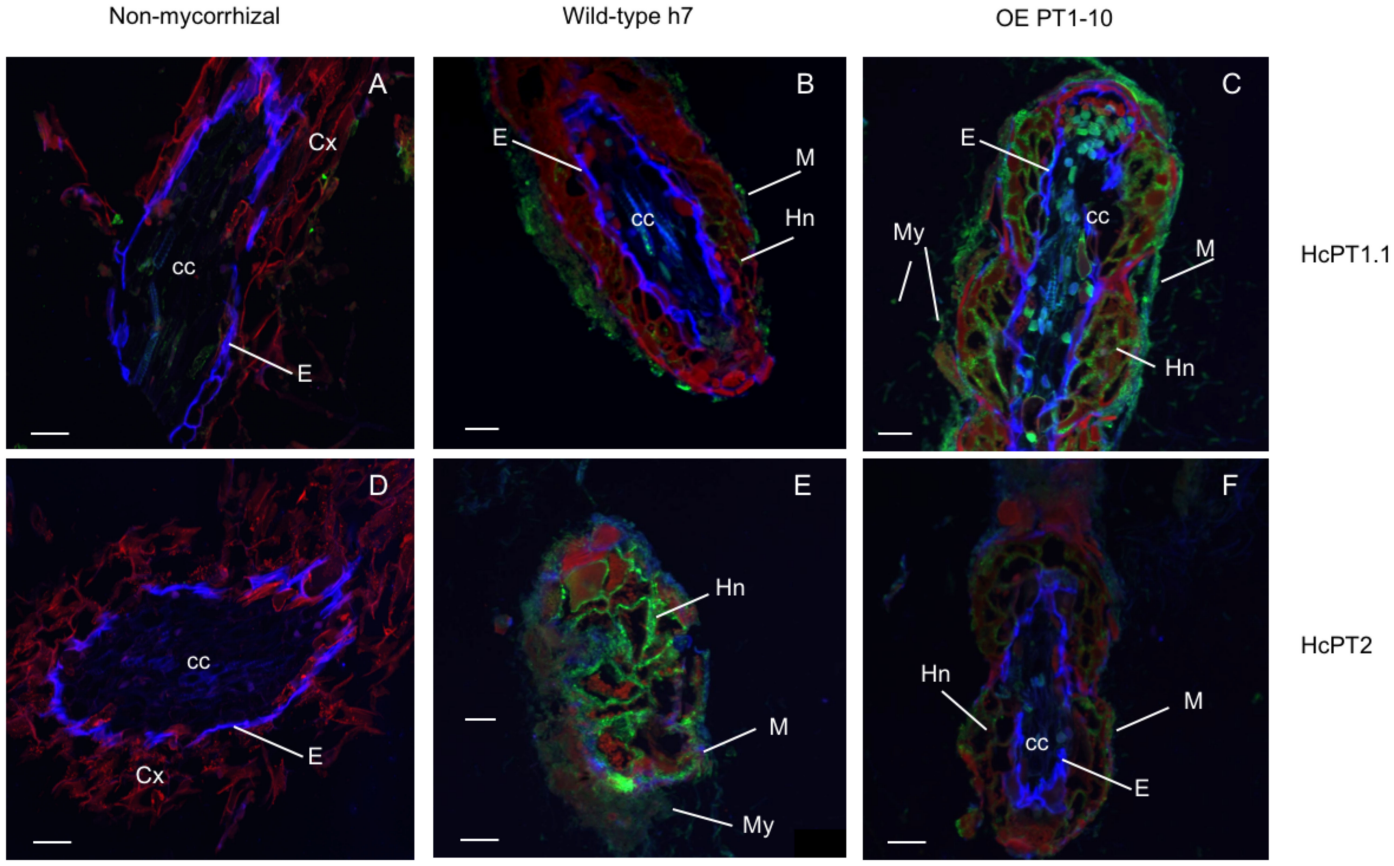
Merged images of root sections probed with the primary antibodies, either anti-HcPT1.1 **(A–C)** or anti-HcPT2 **(D–F)** and the secondary antibodies (Alexa Fluor 488). Images were taken with three fluorescence filters (DAPI, GFP and Rhodamine). The Caspary band delimiting the endodermis autofluoresces in blue (DAPI filter), the signal associated with HcPT1.1 and HcPT2 fluoresces in green (GFP filter) and the root tissues autofluoresce in red (Rhodamine filter). *P. pinaster plants* were grown in limiting-P soil and were either non-mycorrhizal **(A, D)** or associated with the wild *H. cylindrosporum* strain h7 **(B, E)** or the transgenic isolates overexpressing *HcPT1.1*, OE PT1-10. **(C, F)**. M: fungal mantle, My: mycelium, Hn: Hartig net, E: endodermis, cc: central cylinder, Cx: root cortex. Scale bars correspond to 50 µm.

**Figure 5:**
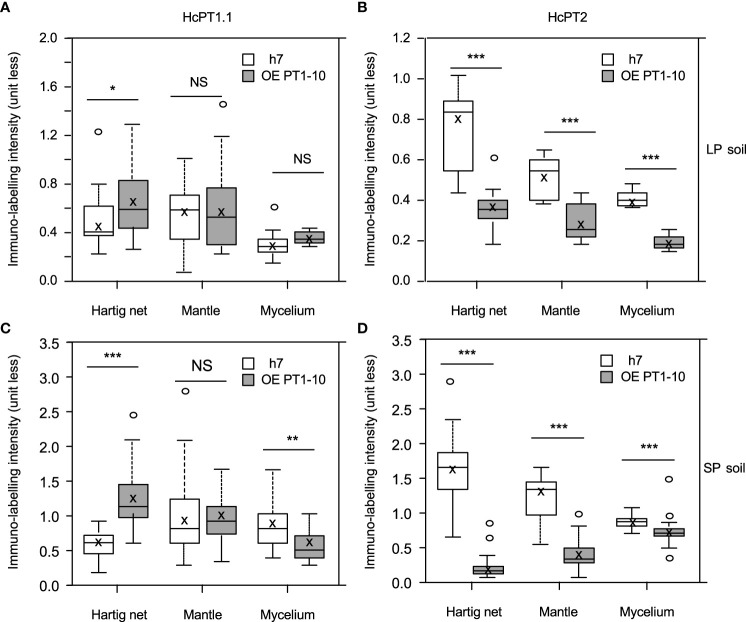
Box plots of relative intensity of immunolabelling of HcPT1.1 **(A, B)** or HcPT2 **(C, D)** measured in sections of ectomycorrhizal roots of *P. pinaster* associated with the wild strain h7 of *H*. *cylindrosporum* or the transgenic line overexpressing *HcPT1.1* (OE PT1-10). Ectomycorrhizal roots were picked on *P. pinaster* plants grown in LP **(A, B)** or SP **(C, D)** soil (same plants as in **Figure 2**). In each box, the cross indicates the mean and the line the median, the box the 1st and 3rd quartiles, and bars the smallest and highest values of relative intensities calculated for each territory (Hartig net, Mantle, and Mycelium) delimited as regions of interest in the images (**Supplementary Figure 3**). For each transporter, significant differences between means of Hartig net, mantle, and mycelium in both fungal lines were analyzed using a Student-t-test (30 < n < 60) and are denoted as follows: NS not significantly different, *p ≤ 0.05, **p < 0,01, ***p < 0.001.

The protein distribution of both fungal H^+^:Pi symporters was quantified using antibodies whose specificity was checked using *S. cerevisiae* strain EY917 (Wykoff and O’Shea, 2001). As shown in **Figure 3A** and 3B, EY917 cells supplied with galactose and uracil grew slowly but decreased the Pi concentration in the medium very quickly because they were expressing their own H^+^:Pi transporter ScPho84. Yeasts expressing *HcPT1.1* or *HcPT2* were able to grow with glucose as a C source (**Figure 3A**; **Supplementary Figures 5A, B**). They were also able to reduce Pi concentration in the medium (**Figure 3B**; **Supplementary Figures 5C, D**), indicating that *HcPT1.1* and *HcPT2* genes encode H^+^:Pi influx transporters. We used one line of each transformant and EY917 to check the specificity of the primary antibodies raised against HcPT1.1 and HcPT2. As shown in **Figure 3C** and **Supplementary Figure 6**, anti-HcPT1.1 or anti-HcPT2 antibodies revealed only the corresponding protein, confirming their specificity. These antibodies were applied on sections of non-colonized short roots grown in LP soil. No staining was visible after immunolabelling with HcPT1.1 or HcPT2 antibodies (**Figures 4A, D**), indicating that they did not target any plant proteins. We immunolocalized HcPT1.1 and HcPT2 in the three ECM pseudo-tissues (*i.e.* extraradical hyphae, mantle, and Hartig net) of 20 ectomycorrhizae collected from the same plants grown in LP and SP soils. Since h7 and Ctl lines, on one hand, and both *HcPT1.1* overexpressing lines, on the other hand, behaved similarly regarding their effect on plant P status (**Figure 2**), the experiment was performed on h7 and OE PT1-10 ECM tips only. The type of fungal isolate had a clear effect on the distribution of the two proteins, HcPT1.1 and HcPT2. In ECM sections produced on LP soil, the protein HcPT1.1 was much more abundant in OE PT1-10 ectomycorrhizae compared to the h7 ones (**Figures 4B, C**). Additionally, the signal for HcPT1.1 seemed to be more localized in the external fungal mantle than in the Hartig net in h7 ECM tips (**Figure 4B**), whereas in OE PT1-10 root tips, the signal was found similarly in the fungal mantle and the Hartig net (**Figure 4C**). The reverse situation was observed for HcPT2, whose signal seemed to be greater in h7 ECM tips than in OE PT1-10 ECM tips (**Figures 4E, F**). These visual observations were confirmed by a more robust quantification of the fluorescent signal on the obtained cross-sections (**Figure 5**). However, the signal from HcPT1.1 antibodies was much more variable than from HcPT2 antibodies, as indicated by the large variations of the relative intensities calculated from the fluorescent pixels for both fungal lines and both soil P conditions (**Figures 5A, C**). Despite these large variations, the HcPT1.1 signal was significantly higher in the Hartig net of OE PT1-10 ECM than h7 ECM tips, especially in SP soil. In the other parts of the ectomycorrhizae, it was either not significantly different in the mantle in both P soil conditions and in the external mycelium of plants grown in LP soil or slightly higher in the mycelium of h7 ECM tips than in OE PT1.1 ones.

The signal from HcPT2 antibodies was less variable than HcPT1.1 (**Figures 5B, D**). In ECM tips associated with h7, the highest levels of HcPT2 signal were found in the Hartig net, whatever the soil P conditions. The signal was lower in the mycelium than in the mantle, also in both soil P conditions. Interestingly, despite these same trends of variation, the values of immunolabelling intensities were about twice as high in SP soil than in LP soil, regardless the part of ectomycorrhiza section examined (**Figures 5B, D**). Remarkably, ECM roots associated with OE PT1.10 always displayed lower levels of HcPT2 signal than h7 ECM tips, regardless of the part of the mycorrhizal root considered or the soil P conditions (**Figures 5B, D**). The most intense decrease in the HcPT2-related signal was localized in the Hartig net of ectomycorrhizae grown in SP soil. Indeed, the signal was around 8 (SP soil) and 2 (LP soil) times lower than the one calculated in h7 ECM tips. In the other ectomycorrhiza parts, immunolabelling intensities in the mantle and the mycelium were half as high as those in h7 ECM tips in LP soil. In SP soil, the signal was 3 (mantle) and 1.3 (mycelium) times lower than the one calculated in h7 ectomycorrhizae.

### 3.4 Effect of *HcPT1.1* overexpression on ^32^P efflux from fungal cells

We previously showed that the overexpression of *HcPT2* in *H. cylindrosporum* mycelia increased ^32^P efflux from fungal cells when incubated with *P. pinaster* compared to mycelia transformed with the control vector (Becquer et al., 2018a). We used the same experimental setup to quantify the effect of HcPT1.1 overexpression on ^32^P uptake and release capacities of *H. cylindrosporum* mycelia. Compared to the wild type (h7) or control vector (Ctl) transformed line, the overexpression of *HcPT1.1* did not significantly alter the mycelial growth and ^32^P uptake in these culture conditions (**Table 1**).

**Table 1:** Fresh weight and ^32^P uptake by the mycelia of *Hebeloma cylindrosporum* either wild-type (h7) or harboring the empty vector (Ctl), or the HcPT1.1-overexpressing (OE-PT1-9 and OE-PT1-10) construct.

	Fungal strain				
	h7	Ctl	0E PT1.9	0E PT1.10	*P*
Growth (g FW)	0.15 ± 0.02	0.16 ± 0.04	0.13 ± 0.03	0.14 ± 0.02	NS
P uptake (µmoles g^-1^ fungal FW)	6.2 ± 1.1	6.6 ± 1.8	5.8 ± 2.1	6.4 ± 1.3	NS

The phosphorus (P) uptake was measured during the ^32^Pi loading period, which lasted 16 h, and is expressed in micromoles of inorganic phosphate (Pi) per gram of fungal fresh weight. Values are the means ± SD (n = 12). Significant differences between means were established using one-way ANOVA at P < 0.05.

**Figure 6:**
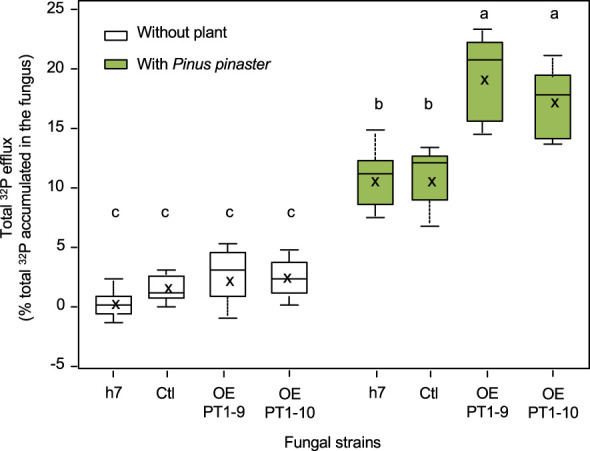
Box plots of total ^32^P efflux (in % of total ^32^P accumulated by the fungus) released by *H. cylindrosporum* lines into the interaction medium and plants during the symbiotic interface-mimicking experiment. The mycelia were incubated alone (without plant) or with plant roots (with *Pinus pinaster*). The fungal lines were the wild-type one (h7) or transgenic ones transformed with the empty vector (Ctl) or overexpressing *HcPT1.1* (isolates OE PT1-9, OE PT1-10). In each box, the cross indicates the mean (n = 6) and the line the median, the box the 1st and 3rd quartiles, and bars the smallest and highest values of ^32^P efflux. Different letters indicate significant differences between the means according to one-way ANOVA followed by Tukey’s HSD test at *P* < 0.05.

^32^P-labelled mycelia were then incubated for 48 h in a P-free and C-free medium buffered at pH 5.9, either alone or with *P. pinaster* roots. Radioactivity was measured in the medium and in the plants, and ^32^P fluxes were normalized by the initial amount of ^32^P accumulated in the mycelia to enable comparisons between lines. As shown in **Figure 6**, an efflux of ^32^P was recorded for all strains. This efflux represented less than 5% of ^32^P in lines incubated without the plants, with no significant differences between fungal lines. The presence of the plant always increased the total ^32^P efflux, plants being the main sink for ^32^P as they accumulated 75% ± 12% of the total ^32^P released by the mycelia, regardless of the line examined. However, both strains overexpressing *HcPT1.1* released significantly more ^32^P than both the wild type (h7) or control vector (Ctl) transformed line when plants were present in the medium (**Figure 6**).

## 4 Discussion

The protein HcPT1.1 of *H. cylindrosporum* was characterized as a H^+^:Pi symporter in yeast and was found to be highly expressed in the external hyphae of ectomycorrhizal roots, especially under low P availability (Tatry et al., 2009). Such features suggest a main role in Pi uptake from the soil into the fungal cells associated with plant roots. However, the protein was also found in the hyphae of the Hartig net (Garcia et al., 2013), a location that raises questions about the actual role of HcPT1.1 in symbiotic P transport. In the present work, we used *HcPT1.1-* overexpressing lines to improve our understanding of the role of this transporter in P movements upon symbiosis.

### 4.1 P accumulation in roots colonized by *HcPT1.1*-overexpressing lines suggests a retention in fungal tissues

Since *HcPT1.1* expression level greatly increases under P-deficiency (Tatry et al., 2009; Garcia et al., 2013), we examined whether its artificial overexpression would improve P allocation to *P. pinaster*, whatever the external P availability. Our results revealed that the roots of *P. pinaster* seedlings associated with these *HcPT1.1* -overexpressing lines accumulated more P than those associated with the corresponding control isolates in LP soil only. Similar observations were already made in plants associated with *HcPT2* -overexpressing transgenic lines (Becquer et al., 2018a). This increase of the absolute amount of total P in ECM roots compared to NM plants is probably due to the presence of fungal cells that were not separated from the root systems during the harvest. Consequently, it is impossible to discriminate between the plant and fungal origin of P detected in roots of ECM plants. Therefore, the higher amount of P observed in roots associated with OE -PT1-9 and OE -PT1-10 lines compared to roots colonized by the control ones could be due to an improved capacity of the *HcPT1.1* -overexpressing fungi to acquire P in P-depleted conditions. Indeed, this higher P content in the fungus could be the result of a cumulative effect involving the artificial overexpression of *HcPT1.1* and its natural upregulation in LP conditions. Also, the main difference with plants associated with OE -PT2 isolates is that here we did not observe any increase of total shoot P contents in the plants grown under both soil P conditions. This lack of P translocation in the plants reinforces the hypothesis of a significant amount of P actually retained in the fungus and not transferred to the host. Because the overexpression of *HcPT1.1* did not affect the expression level of *HcPT2* in pure culture, we could assume the expression level of *HcPT2* in ECM roots is similar between plants associated with the various wild-type and transgenic isolates. Consequently, we decided to examine the distribution of HcPT1.1 and HcPT2 proteins in ectomycorrhizae obtained from plants colonized by the wild-type strain h7 and the overexpressing isolate OE PT1-10 to determine if anything at the protein level could explain this lack of P transport in the plant.

### 4.2 Within ectomycorrhizae, *HcPT1.1* overexpressing lines strongly impacted the distribution of HcPT1.1 and HcPT2 proteins

The localization patterns of HcPT1.1 in h7 ECM root tips confirm our previous observations on HcPT1.1 proteins being more abundant in the fungal mantle than in the Hartig net (Garcia et al., 2013). However, the signal measured in ECM sections obtained from plants grown in LP and SP soils were of the same intensity in the Hartig net and the mantle, contrary to the data of Garcia et al. (2013). This discrepancy might be explained by a decrease of available Pi in SP soil used to supply the plants, which accumulated roughly twice as much as P in SP soil than in LP soil. Hence, at the time of the harvest, the SP soil may have had the same Pi availability than the LP soil, resulting in the enhancement of HcPT1.1 in ECM roots. In contrast to HcPT1.1, the pattern of HcPT2 distribution in h7 ECM root tips is fully in agreement with our previous studies (Tatry et al., 2009; Becquer et al., 2018a), with a greater signal in the Hartig net compared to the mantle and the external mycelium, whatever the external P availability. Also, the signal was much more intense in the Hartig net of ECM roots grown in SP soil than in LP soil, in agreement with the role of this transporter for P movement towards colonized root cells, as proposed by Becquer et al. (2018a).

Immunolabelling also reveals that our agrotransformation strategy significantly increased the signal of HcPT1.1 labelling in ECM root tips formed by the OE PT1-10 isolate compared to the h7 ones, especially in the Hartig net and regardless of the soil P status. Additionally, we observed that the overexpression of *HcPT1.1* negatively altered the distribution of HcPT2 proteins in ectomycorrhizae, particularly in the Hartig net, compared to h7-formed ectomycorrhizae. These observations might be the result of a change in localization, in trafficking, or in competition for membranes, for example. The question of whether or not such a regulation is specific to overexpression or even depends on the host plant remains open.

### 4.3 Do fungal H^+^:Pi symporters have a role in P movement in ectomycorrhizal roots?

The identification of molecular players involved in Pi efflux from the fungus to colonized roots in ectomycorrhizae remains challenging due to the difficulty of investigating the symbiotic interface. P transporters identified in the genomes of ECM fungi could be seen as putative candidates to fulfill this role, including orthologs of *S. cerevisiae* high-affinity (ScPho84/89) and low-affinity (ScPho87/90/91) Pi transporters (Nehls and Plassard, 2018; Plassard et al., 2019). In the ECM symbiosis, most available data on P transporter regulation upon symbiosis concerns H^+^:Pi transporters, homologs to HcPT1.1, HcPT1.2, and HcPT2 (Tatry et al., 2009; Wang et al., 2014; Zheng et al., 2016; Becquer et al., 2018a; Becquer et al., 2018b; Paparokidou et al., 2021). Interestingly, in the *H. cylindrosporum*/*P. pinaster* ECM model, *HcPT2* is the most transcribed P transporter in ectomycorrhizae compared to the free-living mycelium (Doré et al., 2017; Becquer et al., 2018a). It is therefore tempting to hypothesize that HcPT2 may be involved in both the absorption of Pi from the soil by extraradical hyphae and its release towards the symbiotic interface from the Hartig net, as modelized by Schott et al. (2016) and suggested by Becquer et al. (2018a). Here, our results on the distribution of HcPT1.1 and HcPT2 in roots associated with the *HcPT1.1*-overexpressing lines suggest that the decrease of HcPT2 in ECM root tips might be partially compensated by the increase of the HcPT1.1 in the Hartig net. Although it has not been possible so far to measure a P efflux through HcPT2 or HcPT1.1 expressed in yeast, we showed that the overexpression of these two transporters in the hyphae led to a similar increase of ^32^P unloading from the hyphae only in the presence of the host plant (this study, Becquer et al., 2018a). These results suggest that these two H^+^:Pi symporters could have a similar role in P release from the fungal cells under the influence of plant roots. This functional redundancy between the two transporters would explain the maintenance of P status in the shoots of plants associated with *HcPT1.1*-overexpresssing lines grown in SP soil. In this case, *HcPT1.1* was overexpressed in the Hartig net of ectomycorrhizae formed by OE-PT1-10 compared to h7 ones. In contrast, HcPT1.1 was only slightly more abundant in the Hartig net of OE-PT1-10 ECM sections compared to h7 ones in plants grown in LP soil. This slight increase in HcPT1.1 may not have been sufficient to compensate for the strong decrease in HcPT2 proteins, resulting in P over-accumulation in the roots of plants associated with the two HcPT1.1-overexpressing lines. However, this functional redundancy among HcPT1.1 and HcPT2 H^+^:Pi symporters would explain the lack of homologs of HcPT2 found in some fungal genomes, such as in the AM fungus *Rhizophagus irregularis* (Plassard et al., 2019).

However, the mechanisms that could mediate Pi efflux *via* H^+^:Pi symporters such as HcPT1.1 or HcPT2 remain to be elucidated. Indeed, although the results of Fristedt et al. (1996) demonstrated that the high-affinity transporter ScPho84 can mediate Pi transport in both directions, it remained dependent on H^+^ gradients between the two compartments of the vesicle membrane. Thus, *in vivo*, the fungal cell cytoplasm pH should be lower than in the apoplastic space to trigger Pi efflux into the Hartig net. As the apolastic pH is considered to be acidic (around 5.5, Nehls and Plassard, 2018), this would lead to a strong acidification of the cytoplasm which would be incompatible with cell functioning. Nevertheless, two hypotheses could be proposed to explain Pi efflux *via* H^+^:Pi symporters. As mentioned earlier, the first hypothesis involves a simultaneously function of fungal H^+^:Pi and plant H^+^:sugar transport systems in the Hartig net that would allow the release of P into the apoplast, as modelled by Schott et al. (2016). The second hypothesis involves the conversion of fungal H^+^:Pi symporters, such as HcPT1.1 or HcPT2, are modified to become Pi uniporters under the host plant influence. This conversion could be due to direct protein modifications, such as phosphorylation occurring through post-translational modifications, or indirect modifications *via* an allosteric interaction with another phosphorylated protein, as it has been demonstrated for the conversion of a bacterial lactose:H^+^ symporter into a lactose uniporter (Ye et al., 1994).

Finally, regarding the role of HcPT1.1 in *H. cylindrosporum*, further investigations are still needed, such as with the down-regulation of *HcPT1.1*. Also, it would be interesting to investigate the effects of *H. cylindrosporum* on the expression and the localization of P transporters of *P. pinaster*, as similarly described in poplar associated with the ECM fungi *Paxillus involutus* and *Laccaria bicolor* (Loth-Pereda et al., 2011).

## 5 Conclusion

In wild-type ectomycorrhizae formed by h7 *H. cylindrosporum* and *P. pinaster*, HcPT1.1 mainly participates in P acquisition from the soil under low P, and HcPT2 in its transfer towards the host cells (Tatry et al., 2009; Garcia et al., 2013; Becquer et al., 2018a). However, our results here suggest that HcPT1.1 could have a similar role to HcPT2 upon symbiosis, indicating a possible functional redundancy mechanism and/or tight regulatory interactions between HcPT1.1 and HcPT2 at the protein level. Indeed, it appears that transcriptional regulation, protein abundance, distribution, and functional activity of the subset of fungal Pi transporters are highly linked. It would be interesting to analyze further the limiting steps of such nutrient exchange from the fungus towards the plant. Simultaneous overexpression of both transporters, HcPT1.1 and HcPT2, would be highly interesting to analyze their impact on P transfer.

## Data availability statement

The raw data supporting the conclusions of this article will be made available by the authors, without undue reservation.

## Author contributions

CP, KG and SDZ contributed to conception and design of the study. LA, AB and CT-S acquired the data; LA, AB and CP organized the data. AB and CP performed the statistical analysis. CP and KG wrote the first draft of the manuscript and all authors contributed to manuscript revision, read, and approved the submitted version.
