# Supplementary material for: Overexpression of the HcPT1.1 transporter in Hebeloma cylindrosporum alters the phosphorus accumulation of Pinus pinaster and the distribution of HcPT2 in ectomycorrhizae
Source: Front Plant Sci. 2023 Jun 15;14:1135483. doi: 10.3389/fpls.2023.1135483 (PMC10325726; doi:10.3389/fpls.2023.1135483)
Supplement: Supplementary file 1 [file DataSheet_1.docx]

Supplementary Material

# Supplementary Method 1 - Symbiotic interface-mimicking experiment

*Plant preparation*
Two-month old non-mycorrhizal plants grown in test tubes as described in section 2.3 were used in this experiment. The nutrient solution containing KH_2_PO_4_ 0.2 mM, was replaced by 30 ml sterile CaSO4 (0.2 mM) for 1 week to exhaust soluble orthophosphate. Finally the CaSO4 solution was substituted by an interaction medium (MgSO4 0.2 mM, CaCl2 0.5 mM, TRIS 5 mM, MES 5 mM, pH 5.9 after autoclaving at 120°C, 20 min) for 24 h to acclimatize the roots. These incubations were performed in the growth chamber under conditions described in section 2.3 of the main text.

*Fungal material*

The fungi were grown in 120 ml glass flasks containing 40 ml of N6 liquid medium (6 mM KNO3, 4 mM KCl, 1 mM CaCl2, 1 mM MgSO4, 1 mM NaH2PO4, 50 μg l–1 thiamine hydrochloride, 0.5 ml l–1 1% Fe citrate solution, 0.2 ml l–1 micronutrients of Morizet & Mingeau (1976) solution and 55 mM glucose, pH 5.5; Becquer et al. 2017a) supplemented with carboxin (0.2 mg l-1) for the transgenic lines. The mycelia were grown first for 2 weeks, in dark condition, without shaking. Then, new cultures without agar plug were launched in the same medium, by cutting fungal disks (7.5 mm diameter) directly into the 2-week-old mycelium. After 12 days, the mycelia were transferred into new flasks containing the same solution but without Pi (-P solution) for 7 additional days. The resulting P-starved mycelia were transferred for 16 h into a fresh +P solution containing 1 mM of Pi, labelled with 32P (P-resupply period).

*Incubation*
After 16 h of P-resupply labelled with 32P, mycelia growing in flasks were suspended into the syringes filled with 15 ml of interaction medium. Mycelia were rinsed 5 times during 1, 15, 30, 45 and 90 min with 15 ml of fresh interaction medium. Finally, the syringes were filled with 60 ml of fresh interaction medium (0 h) and fungi were incubated alone or with or without the roots of two 2 month-old *P. pinaster* seedlings under continuous light for 48 h (Becquer et al. 2017b, 2018).

*32P labelling and calculations relative to 32P data*

The nutritive solution used for 32P loading into the P-starved mycelia was made from the +P solution supplemented with radiolabeled phosphorus as dipotassium phosphate (specific activity of 900-1100 mCi/mmol, ref NEX055002MC) from Perkin-Elmer. The radiolabeled source was diluted to obtain an initial activity of 3.3 × 104 Bq per μmol Pi in the solution. The actual labelling of the solution used to load the mycelia and the radioactivity released from the fungal material into the interaction medium were measured by mixing respectively 0.05 ml of the nutrient solution or 1 ml of induction medium with 4 ml of scintillation cocktail in a 6 ml- scintillation vial. For solid samples, 5 ml (mycelia in 6 ml-scintillation vials) or 15 ml (plants in 20 ml-scintillation vials) of scintillation cocktail were added to the vial to cover the tissues. The vials were shaken vigorously before radioactivity was measured in a scintillation counter (Liquid scintillation Counter TRI-CARB 4910TR).

Raw data were corrected for decay occurring during radioactivity measurement using the following equation written in Excel :

cpmt0 = Exp(Ln(cpmt-cpmbackground)) + (0.00003366099 x t),

with cpm = counts per minute

t0 = time of the beginning of experiment
t = time of cpm measurement of the sample in counter in minutes, cpmbackground = cpm of solvent (blank sample),
cpmt = radioactivity of the sample at time t,

cpmt0 = radioactivity of the sample at time t0 corresponding to the first measurement. This value was taken as the reference one for all measurements and all values are converted from cpm to Bq (Becquerel) by multiplying the number of cpm by 0.033.
The specific activity (SAi) of phosphate supplied to the fungus (replicatei) was calculated for each replicate as follows, assuming that the concentration of phosphate is 1 mM:

SAi (Bq/μmole Phosphate) = [(cpmt0 of labelled medium/0.05 (ml) x 0.033].
Total 32P released in the IM was obtained by multiplying the activity of IM (in Bq ml-1) with the volume recorded at 48 h in the corresponding syringe.
The total amount of 32P accumulated in the fungus at time 0 of incubation with or without plant was calculated by summing the amount of 32P accumulated in the mycelium and the amount of 32P released into the induction medium when a mycelium was incubated alone; and the amount of 32P measured in plants when the mycelium was incubated with the plant.
Finally, the amount of P taken up by the fungusi (replicate i) during the exposure to 32P and at the beginning of incubation was calculated as follows:
P accumulation in fungusi (μmoles) = (Bq in the fungusi) / SAi

**References cited**

Becquer, A., Garcia, K., Amenc, L., Rivard, C., Doré, J., Trives-Segura, C., Szponarski, W., Russet, S., Baeza, Y., Lassalle-Kaiser, B., Gay, G., Zimmermann, S.D., Plassard, C., 2018. The *Hebeloma cylindrosporum* HcPT2 Pi transporter plays a key role in ectomycorrhizal symbiosis. New Phytologist 220, 1185–1199. doi:10.1111/nph.15281

Becquer, A., Torres-Aquino, M., Le Guernevé, C., Amenc, L.K., Trives-Segura, C., Staunton, S., Quiquampoix, H., Plassard, C., 2017a. A Method for Radioactive Labelling of *Hebeloma cylindrosporum*  to Study Plant-fungus Interactions. Bio-Protocol 7, e2576. doi:10.21769/BioProtoc.2576

Becquer, A., Torres-Aquino, M., Le Guernevé, C., Amenc, L.K., Trives-Segura, C., Staunton, S., Quiquampoix, H., Plassard, C., 2017b. Establishing a Symbiotic Interface between Cultured Ectomycorrhizal Fungi  and Plants to Follow Fungal Phosphate Metabolism. Bio-Protocol 7, e2577. doi:10.21769/BioProtoc.2577

Morizet, J., Mingeau, M., 1976. Influence des facteurs de milieu sur l’absorption hydrique. Etude effectuee sur tomate decapitee en exsudation. i. facteurs nutritionnels. Ann. Agron. 27, 333–342.

# Supplementary Figures and Tables

## Supplementary Figures


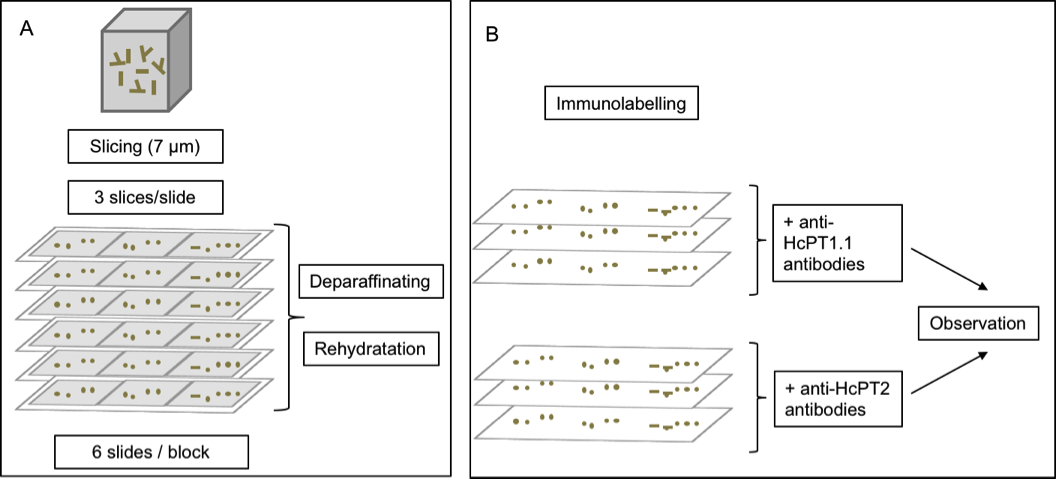


**Supplementary Figure 1.** Method description for the preparation of root sections immunolabelled with antibodies against HcPT1.1 or HcPT2. Step A: for each treatment (soil condition x mycorrhizal treatment), one paraffin block containing about 10 root tips was cut using a microtome to generate 7 µm thick slices that were layered on 6 glass slides; the slides were then deparaffinated and rehydrated. Step B: the slides were immunolabelled with antibodies anti-HcPT1.1 or anti-HcPT2 and revealed with AlexaFluor 488.


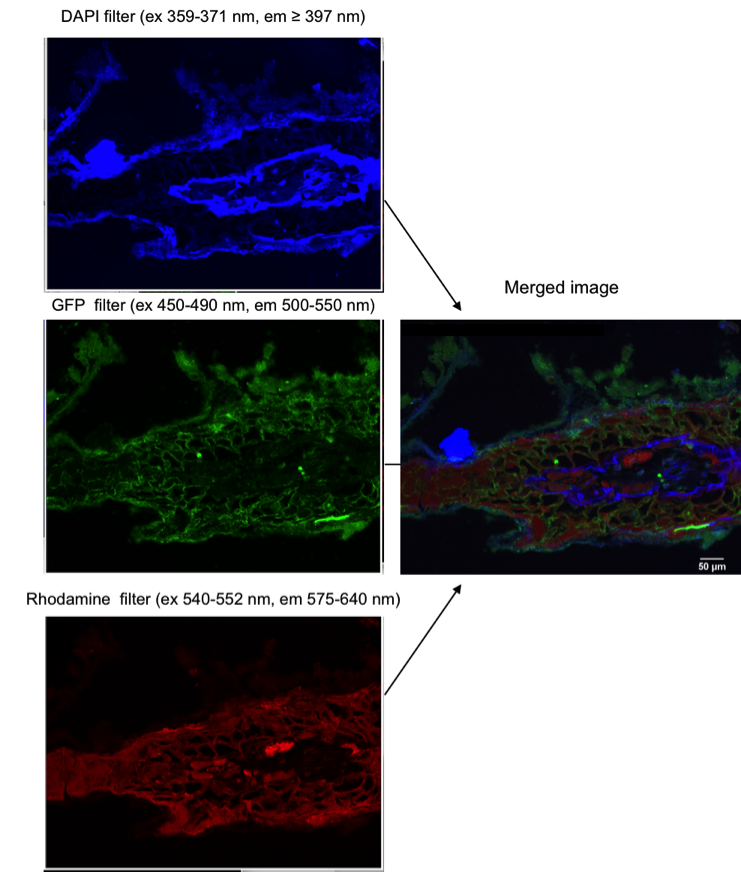


**Supplementary Figure 2.** View of images from an ECM section using an epifluorescence microscope taken with the three filters (DAPI, GFP and Rhodamine) recorded independently or merged together.


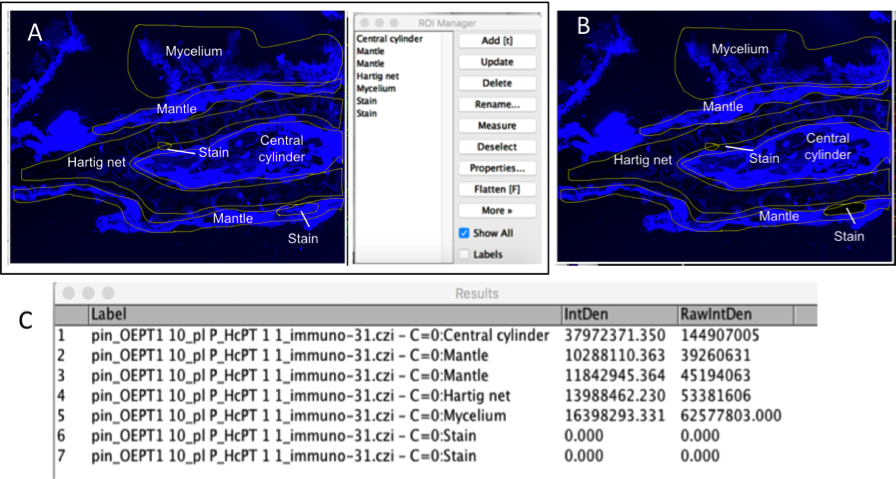


**Supplementary Figure 3**. Method of image analysis developed in ImageJ to record pixel intensities of functional zones of ECM sections. A/ The first image given in Figure S1 (DAPI filter) was visually inspected to define the different ROI (Region Of Interest) zones which are central cylinder, mantle, Hartig net, and mycelium. In case of abnormal fluorescence appears in the image of the GFP filter, they were also defined as “Stain”. Each zone was successively added in the ROI manager of ImageJ. B/ Each stain zone was suppressed from the image. C/ Pixel intensities for each zone were recorded from the image using the function “Measure” in ImageJ. ROI zones were then applied to the two other images (GFP and Rhodamine filters in Supplementary Figure 1) to extract the corresponding pixel intensities from each zone.


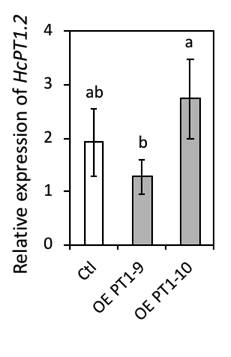


**Supplementary Figure 4.** Expression levels of *HcPT1.2* in *HcPT1.1*-overexpressing lines of *H. cylindrosporum* grown in pure culture. Gene expression was quantified using qRT-PCR in fungal lines transformed with the empty vector (Ctl) and *HcPT1.1* (OE-PT1.1-9 and OE-PT1.1-10). Relative expression was normalized using the α-tubulin housekeeping gene from *H. cylindrosporum*. Bars correspond to mean values ± SD (n=4). Different letters indicate significant differences between means according to one-way ANOVA followed by Tukey’s HSD test (P < 0.05).


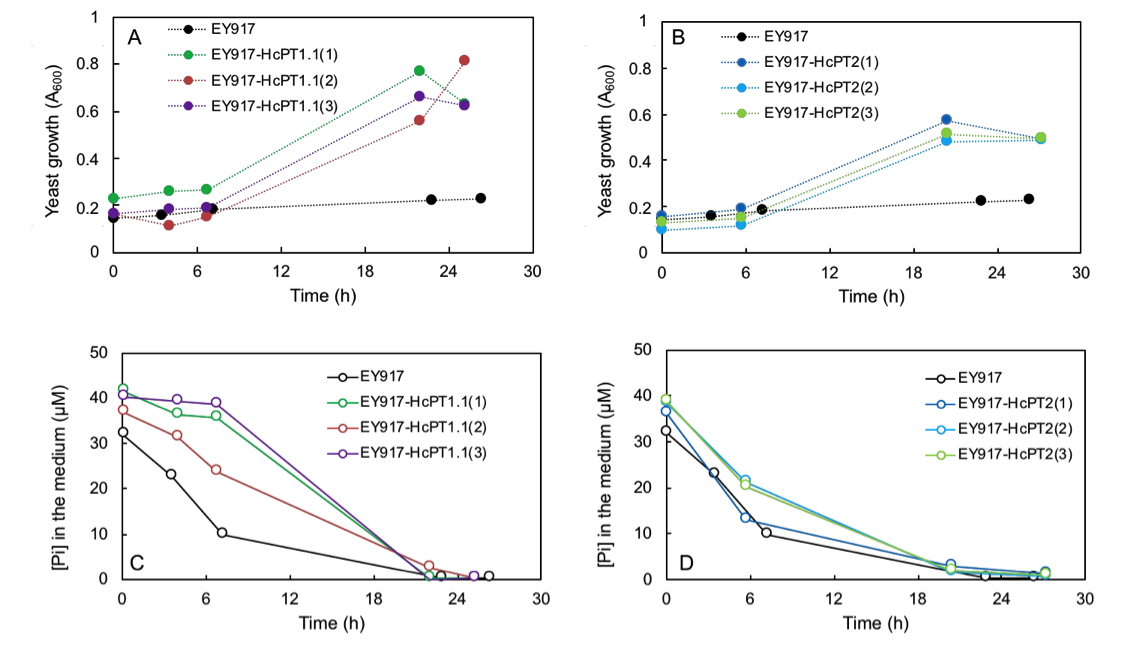


**Supplementary Figure 5.** Growth curves (A, B) and Pi depletion (C, D) from the medium following culture of *Saccharomyces cerevisiae* strain EY917 expressing *ScPho84* (EY917), or three transformants expressing *HcPT1.1* (EY917-HcPT1.1) (A, C) or *HcPT2* (EY917-HcPT2) (B, D). The yeast EY917 was grown with galactose and uracil to express *ScPho84*, whereas transformants expressing *HcPT1.1* or *HcPT2* were grown with glucose, without uracil.


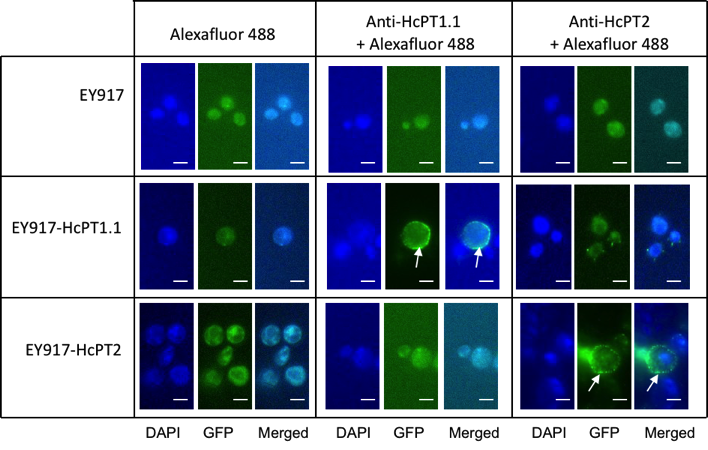


**Supplementary Figure 6.** Immunolocalisation of HcPT1.1 or HcPT2 in *Saccharomyces cerevisiae* strain EY917 expressing *ScPho84* (EY917), *HcPT1.1* (EY917-HcPT1.1) or *HcPT2* (EY917-HcPT2). The yeasts were probed either with the secondary antibody only (Alexa Fluor 488) or the primary antibodies, either anti-HcPT1.1 or anti-HcPT2, together with Alexa Fluor 488. Images were taken with DAPI and GFP filters. and merged together. Immunolabelling revealed a plasma membrane localisation of HcPT1.1 or HcPT2 (white arrows) only in the yeast transformed with the plasmid harbouring *HcPT1.1* or *HcPT2* cDNA. Scale bars correspond to 5 µm.

## Supplementary Tables

**Supplementary Table 1.** Primers used in this study. Restriction enzyme sites, indicated in primer name, were underlined in the corresponding 5’ – 3’ sequence.

| Experiment/ Construct | Name | 5’-3’ sequences |
| --- | --- | --- |
| EY917- *HcPT1.1* | HcPT1.1-For  pFL61-Rev | CGGCTTCAGTCAGCTCAAGGATATTGGTGGC  CTGCATAAAGGCATTAAAAAGAGGAGCG |
| EY917- *HcPT2* | HcPT2-For  pFL61-Rev | GCGGCAGCTGGAAAGGCTGGTGCTATC  CTGCATAAAGGCATTAAAAAGAGGAGCG |
| OE*-HcPT1.1* | HcPT1-SpeI-For | CCTTACTAGTATGGCTAGCTACCAAGAGAAAGG |
|  | HcPT1-SpeI-Rev | CCTTACTAGTTTAAGCGGTGGTGATGGGCGCA |
| *HcTub*-qPCR | HcTub-769-QF | ACCTGGTTCCTTTCCCTCGA |
|  | HcTub-944-QR | GCGCATGCCATGTATTTTCCT |
| *HcPT1.1*-qPCR | qPCR-PT1.1-Q1For | CACAAATAAATTCGTCAAGCATATTCTCG |
|  | qPCR-PT1.1-Q1Rev | GCGTTCTCGCACACCTCTG |
| *HcPT2*-qPCR | qPCR-PT2-Q2For | CTTCGGTTGCTGTATCGCTG |
|  | qPCR-PT2-Q2Rev | TACGCACACGGATTTCCTCC |
| *HcPT1.2*-qPCR | HcPT1.2-1447-F3For | CATTATGGCCGGAGCGTAC |
|  | HcPT1.2-1702-R3 | CTCCGAGAGCGATTTGGATC |

**Supplementary Table 2.** Liquid synthetic medium to follow Pi uptake by the yeasts.

| **Compound** | **Final concentration** | **Sterilization mode** |
| --- | --- | --- |
| Glycerol | 20 g l^-1^ | autoclaving |
| KCl | 10 mM | autoclaving |
| MgSO_4_, 7H_2_O | 2 mM | autoclaving |
| CaCl_2_ | 4.5 mM | autoclaving |
| (NH_4_)_2_SO_4_ | 8 mM | autoclaving |
| KH_2_PO_4_ | 0.03 mM | autoclaving |
| Microelement solution^$^ | 1 ml l^-1^ | autoclaving |
| MES hydrate pH 5 | 25 mM | autoclaving |
| Vitamin solution^$^ | 1 ml l^-1^ | 0.22 µm filtration |
| Amino acids^$$^ | 1.368 g l^-1^ | 0.22 µm filtration |
| L-leucine | 40 mg l^-1^ | 0.22 µm filtration |
| Sugar (galactose or glucose) | 20 g l^-1^ | autoclaving |

^$^: full composition given in Supplementary Table 3; ^$$^: amino acids dropped out L-leucine, L-histidine, L-tryptophan and uracil

**Supplementary Table 3.** Composition of micronutrient and vitamin solutions added in synthetic medium.

| **Micronutrient solution** | |  | **Vitamin solution** | |
| --- | --- | --- | --- | --- |
| **Salt** | **Concentration (g l^-1^)** |  | **Compound** | **Concentration (g l^-1^)** |
| H_3_BO_3_ | 0.5 |  | Ca-panthothenate | 5 |
| CuSO_4_ 5H_2_O | 0.27 |  | Myo-inositol | 20 |
| KI | 1.032 |  | Nicotinic acid | 2 |
| MnSO_4_ H_2_O | 2.0 |  | Pyridoxal HCl | 0.25 |
| NaMoO_4_ 2H_2_O | 0.2 |  | Thiamine HCl | 0.25 |
| Fe_2_(SO_4_)_3_ | 2.6 |  | Biotin | 0.1 |
| ZnSO_4_ 7H_2_O | 0.4 |  |  |  |
| Co(II)Cl_2_ 6H_2_O | 0.12 |  |  |  |
| NiSO_4_ 6H_2_O | 0.1 |  |  |  |
